# Experimental Investigation of the Compactability and Cracking Behavior of Polyacrylamide-Treated Saline Soil in Gansu Province, China

**DOI:** 10.3390/polym11010090

**Published:** 2019-01-08

**Authors:** Tongwei Zhang, Yongfeng Deng, Hengxing Lan, Fanyu Zhang, Huyuan Zhang, Chong Wang, Yu Tan, Rongguang Yu

**Affiliations:** 1 Key Laboratory of Mechanics on Disaster and Environment in Western China, Department of Geological Engineering, College of Civil Engineering and Mechanics, Lanzhou University, Tianshui Road 222, Lanzhou 730000, China; zhangfy@lzu.edu.cn (F.Z.); zhanghuyuan@lzu.edu.cn (H.Z.); wangchong@lzu.edu.cn (C.W.); tany17@lzu.edu.cn (Y.T.); yurg13@lzu.edu.cn (R.Y.); 2 Institute of Geotechnical Engineering, School of Transportation, Southeast University, SiPailou 2, Nanjing 210096, China; 3 State Key Laboratory of Resources and Environmental Information System, Institute of Geographic Sciences and Natural Resources Research, Chinese Academy of Sciences, Beijing 100101, China; lanhx@igsnrr.ac.cn; 4 School of Geological Engineering and Geomatics, Chang’an University, Xi’an 710064, China

**Keywords:** saline soil, polyacrylamide, consistency limits, compactability, desiccation crack behavior

## Abstract

Polyacrylamide (PAM) is a water-soluble polymer with the ability to enhance a soil’s stability. PAM is currently being used to prevent irrigation-induced erosion and enhance the infiltration in farmland soil. To improve the compaction properties of the saline-soil-based filling material that is used in highway subgrade and the cracking resistance capacity of a saline soil’s crust, the consistency limits, compactability, microstructure, and cracking morphology of untreated and PAM-treated saline soil were investigated. The saline soils were sampled from the soil crust and a depth of 2.0–3.0 m in Gansu Province, China. Two PAM concentrations (0.1% and 0.5% in mass ratio) were selected. The liquid limits and plastic limits of the saline soil samples from the surface (0–0.05 m) and a depth of 2.0–3.0 m noticeably increased as PAM concentration increased. The maximum dry densities decreased as PAM concentration and plasticity increased, and the optimum water contents of the two saline soil types did not significantly change. These results suggest that a higher shearing resistance between particles partially prevented compression from occurring during compaction. Mercury intrusion porosimetry (MIP) and scanning electron microscopy (SEM) test results showed that the PAM agent dispersed the bulky pellets, and the soil’s structure was formed by flaky and acicular platelets that filled the micropores. A quantitative analysis of crack patterns showed that the cross-points of the crack network and the crack length decreased as the PAM concentration increased. These results indicate that an increase in PAM reduces the shrinkage strain and the flaws or pores within saline soils. Therefore, PAM’s stabilizing effect on saline soil under a wetting–drying cycle was proven.

## 1. Introduction

Many deposits of quaternary sediments, such as saline soil, exist in Western China and increasing soil salinity is a serious land degradation issue around the world. Excessive amounts of salts have adverse effects on the physical and chemical properties of soil, such as soil collapse, erosion, swelling, and desiccation cracks [1]. Therefore, improving the compaction properties and cracking resistance capacity of the saline-soil-based filling material that is used in highway subgrade is an important geotechnical engineering issue.

The use of polyacrylamide (PAM) in soil conditioning began in the 1950s. The concept that anionic polymers can flocculate aqueous clay suspensions was first introduced by Ruehrwein and Ward (1952) [2], who proposed that a single polymeric chain could attach itself to more than one particle’s surface. Since then, polymeric-based additives have been demonstrated to reduce permeability and increase durability, and are not time-dependent during the mixing stage [3,4,5,6]. PAM is a water-soluble polymer with the ability to enhance a soil’s stability. It is currently being used to prevent the irrigation-induced erosion and enhance the infiltration of farmland soil [7,8,9,10,11]. Construction sites, such as foundations and highway embankments, are also vulnerable to drying-induced soil cracking and aggregate soil stability degradation. Civil engineering has been intensely focusing on the use of PAM as a soil-stabilizing additive. Orts et al. (2007) [10] reported the rapid formation of helicopter landing pads that can minimize the dust clouds that appear during helicopter landings in military operations. Deng et al. (2012) [3] found that a PAM dosage of 0.3% can improve the flexibility of cemented soil by increasing the failure strain by up to 6%. Georgees et al. (2018) [12] conducted unconfined compressive strength (UCS) and repeated load triaxial (RLT) tests on PAM-treated soils. The results showed that the addition of PAM consistently improved the strength properties and resilience moduli of the tested soils. Although some physicochemical properties of PAM-stabilized soils have been studied, data for the compactability and cracking behavior of PAM-stabilized saline soil from Gansu Province, China are lacking.

In this study, saline soil was sampled from the crust and at a depth of 2.0–3.0 m in Gansu Province, China. Then, the basic physical properties, compactability, microstructure, and cracking morphology of untreated and PAM-treated soil samples were investigated. Two PAM concentrations (0.1% and 0.5% mass ratio) were selected. First, consistency tests were conducted by mixing saline soil samples with PAM solutions. Second, proctor compaction tests were performed to determine the soil samples’ compaction curves. After that, their microstructures were investigated using mercury intrusion porosimetry (MIP) and scanning electron microscopy (SEM) tests. Using an image-processing technique, surface crack lengths were characterized quantitatively after desiccation tests. Finally, the correlations among the obtained results were identified.

## 2. Experimental Methods

### 2.1. Materials

**Figure 1:**
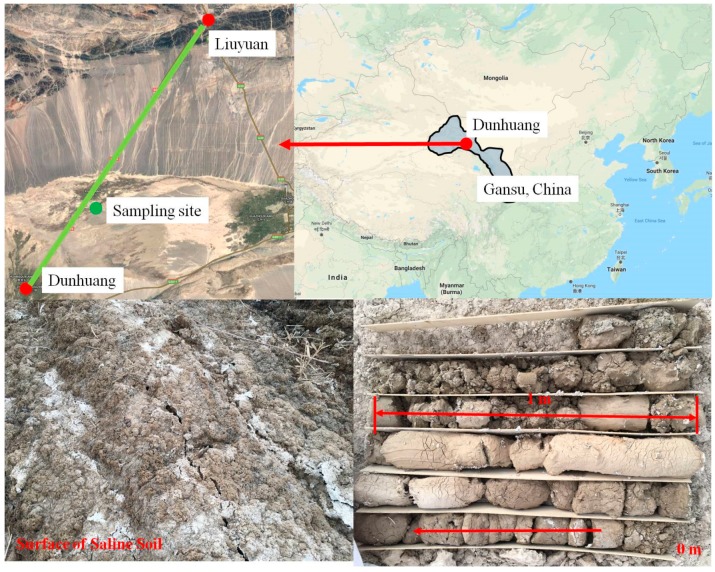
Sampling site and the appearance of saline soil on the surface and at a depth of 2.0–3.0 m.

**Figure 2:**
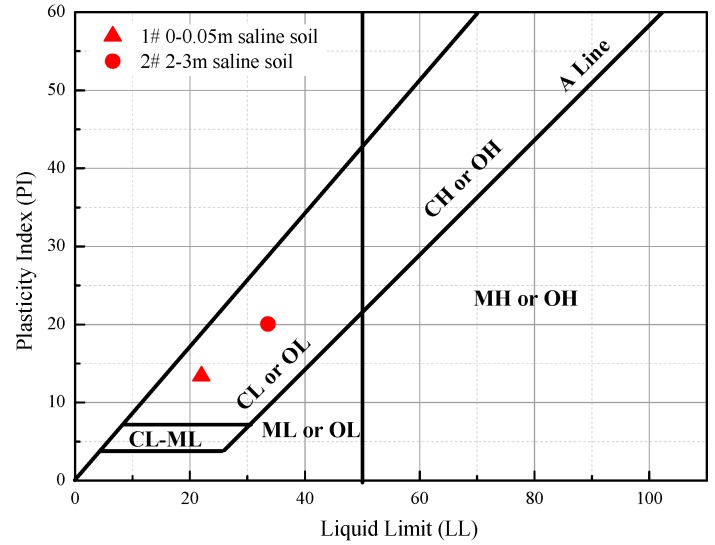
Plasticity chart for Sample #1 and Sample #2. (CL is lean clay, CH is fat clay, OL is organic silt, OH is organic silt, ML is silt, MH is elastic silt. See more details in American standard D2487).

The Dunhuang region is located in the western section of Hexi Corridor in Gansu Province, China. Saline drylands cover a large area of this region. The water table lies between 2 and 3 m from the surface of the soil. As shown in Figure 1, the saline soil samples were obtained near a highway (from Liuyuan to Dunhuang) embankment in Dunhuang at depths of 0–0.05 m and 2.0–3.0 m. Soil salinization was observed on the surface, and the lower-depth soil was soft and had a high water content. The soil’s basic physical properties, such as the natural water content *w*_0_, the dry density ρ_d_, and the compression coefficient *a*, are shown in Table 1. The results show that the in-situ water content, void ratio, and compressibility of the soil at a depth of 2–3 m were higher than those of the surface soil. The ion concentrations, which are shown in Table 2, were determined in accordance with Chinese standard GB/T 50123. The results show that the total salinity in the upper soil was 1.53% as compared to 0.99% in the lower soil. Ca^2+^ and Cl^−^ were found to be the main ions in the surface soil, whereas Na^+^/K^+^ and Cl^−^ were found to be the main ions in the soil at a depth of 2.0–3.0 m. The concentration of the main monovalent cation (Na^+^/K^+^) in the soil at a depth of 2.0–3.0 m was found to be approximately 50 times higher than in the surface soil. The concentration of the main bivalent cation (Ca^2+^) in the surface soil was found to be 15 times higher than in the soil at a depth of 2.0–3.0 m. The liquid limits (LLs) and plastic limits (PLs) of the selected soil samples were determined using the cone method in accordance with GB/T 50123. As shown in Figure 2, based on the United Soil Classification System ASTM-D2487, the tested materials were both classified as low-plasticity clays (CL). A commercial polyacrylamide (PAM) reagent of analytical grade was adopted in this study.

### 2.2. Test Method

**Figure 3:**
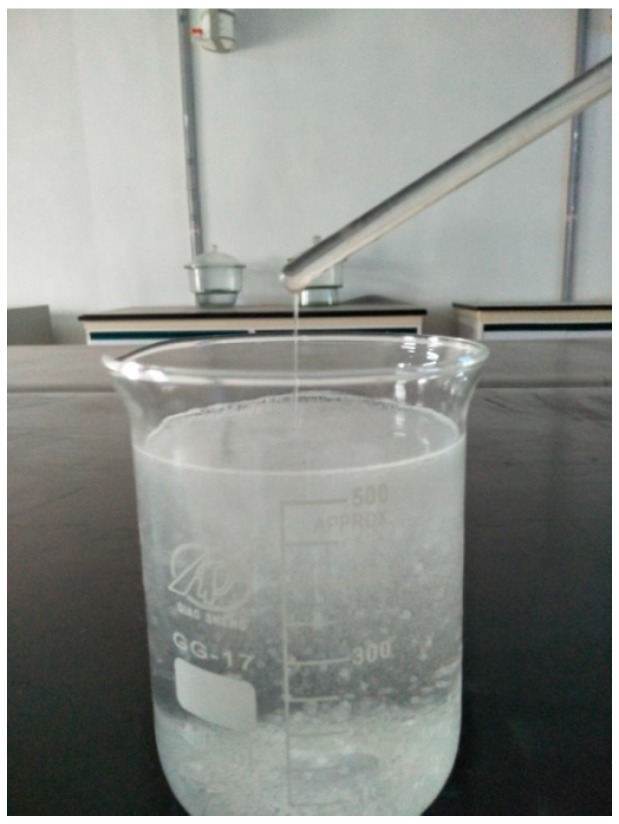
The appearance of the polyacrylamide (PAM) solution, which contains a large amount of suspended flocculates.

To investigate the basic properties of the PAM-treated saline soil samples, consistency tests were conducted by mixing saline soil samples with PAM solutions (the mass concentrations were 0.1% and 0.5%). The test procedures followed Chinese Standard GB/T 50123. During the preparation of the PAM solution, a decrease in the fluidity and an increase in the viscosity were observed, as shown in Figure 3. The reason for this finding is that PAM powder is highly water-absorbent and forms a soft gel when it is hydrated.

The Proctor compaction tests were performed following Chinese standard GB/T 50123. The soil samples were sieved through a 5-mm sieve, then carefully wetted to the required water content and sealed in plastic bags for 24 h. The mold’s diameter was 102 mm. The compaction energy was 592.2 kJ/m^3^. Finally, compaction curves for the water content *w* and the dry density ρ_d_ were obtained.

After the compaction curves were obtained, the microstructures of PAM-treated saline soil samples were investigated using MIP and SEM tests. MIP is a method for determining the pore size distribution of a porous material. It is based on the unique relationship between intrusion pressure and equivalent pore diameter proposed by Washburn (1921) [13] and shown in Equation (1)

(1) $$D=-\frac{4\gamma \cos\theta }{P}$$

where *D* is the pore diameter, *γ* is the surface tension of mercury, *θ* is the contact angle, and *P* is the applied pressure. Based on the contact-angle measurements of mercury with typical clays performed by the previous researchers [14,15,16], a contact angle of 140° and a mercury surface tension of 0.480 N/m were adopted in this study. As the intrusion pressure range of a PoreMaster-60 (Quantachrome Instruments Corporation, Boynton Beach, FL, USA) was 3.7 KPa to 241.1 MPa, the pore sizes measured from 0.005 to 340 μm. To minimize sample shrinkage, small soil specimens were taken from a compacted soil sample, then immersed in liquid nitrogen (−196 °C) to instantly freeze them. Then, the frozen specimens were transferred to the vacuum chamber of a freeze dryer for sublimation for approximately 24 h [17].

To prepare the samples that were used in the SEM tests, compacted soil samples were carefully trimmed to appropriate sizes and then immersed in liquid nitrogen [17]. Finally, they were lyophilized, vacuum-coated with a layer of gold, and installed in the instrument JSM-5600LV (JEOL Ltd., Tokyo, Japan).

The desiccation tests were conducted following the procedure described by Tang et al. (2010) [18]. Specimens were prepared by mixing the dry soil powder (sampled at depths of 0–0.05 m and 2.0–3.0 m) with distilled water or a PAM solution (0.1% and 0.5% in mass ratio) to achieve a higher water content than the liquid limit (*w*_0_ = 75%). The mixtures were thoroughly stirred for 10 min, and then a quantity of the soil-water slurry was slowly poured into a round glass container with a 120–mm diameter. To homogenize the slurry’s density, the containers were vibrated for at least 5 min. All samples were placed in an oven at 50 ℃. During the drying process, the water loss was tracked by weighting each specimen, and a digital camera D750 (Nikon Co., Tokyo, Japan) was used to monitor the evolution of surface cracks.

To quantitatively investigate the soil salinity’s effect on the cracking behavior, digital image-processing techniques were introduced. First, an original photo was transformed to a grey image. Second, the grey image was converted to binary information to separate the clods and cracks. Third, the black parts, which represent a network of cracks, were recognized using the commercial software ‘Image J’ (National Institutes of Health, Research Triangle Region, NC, USA). Finally, the total crack length was quantitatively summarized.

## 3. Results

### 3.1. Consistency Limits of the PAM-Treated Saline Soil Samples

**Figure 4:**
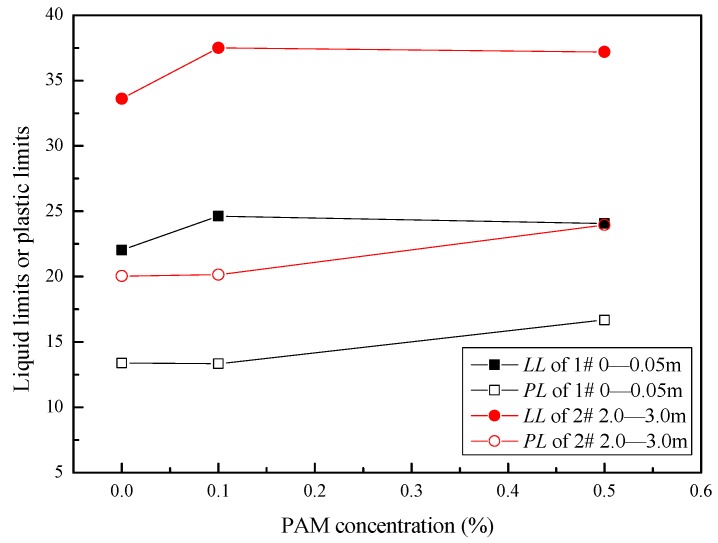
Liquid limits (*LL*) and plastic limits (*PL*) with a changing polyacrylamide (PAM) concentration.

The LL*s* and PL*s* of two PAM-treated soil samples with changing PAM concentrations (0.1% and 0.5% in mass ratio) are shown in Figure 4. The results show that the two saline soil samples behave similarly with respect to the PAM concentration. In the case of Sample #1 (0–0.05 m), as the PAM concentration in the pore water increased, the LLs and PLs increased from 22.0% and 13.4% to 24.6% and 16.6%, respectively. In the case of Sample #2 (2.0–3.0 m), the LLs and PLs increased from 33.6% and 20.0% to 37.5% and 23.9%, respectively, as the PAM concentration increased. Sridharan and Rao (1975) [19] and Sridharan et al. (2002) [20] proposed that inter-particle friction and the absorbed water volume dominate the liquid limit. Thus, these results indicate that the polymer may increase the amount of water that is stored between particles and increase the inter-particle friction.

### 3.2. Compaction Curves for the PAM-Treated Saline Soil Samples

**Figure 5:**
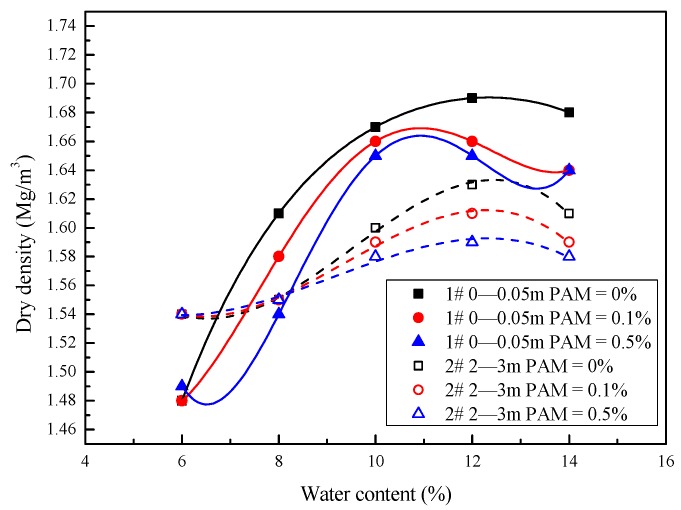
Compaction curves of Sample #1 and Sample #2 treated with polyacrylamide (PAM) solution.

Figure 5 shows the standard Proctor compaction curves for the samples. The maximum dry densities of Sample #1 with distilled water, 0.1% PAM, and 0.5% PAM were 1.69, 1.67, and 1.66 Mg/m^3^, respectively. The maximum dry densities of Sample #2 with distilled water, 0.1% PAM, and 0.5% PAM were 1.63, 1.61, and 1.59 Mg/m^3^, respectively. The maximum dry densities decreased as the PAM concentration and the plasticity increased (the *LL* of Sample #2 was higher than that of Sample #1). Similar trends in experimental results were reported by Blotz et al. (1998) [21]. The optimum water contents of Samples #1 and #2 were 11% and 12%, respectively, and the PAM concentration had no significant influence.

### 3.3. Microstructure of the PAM-Treated Saline Soil Samples

**Figure 6:**
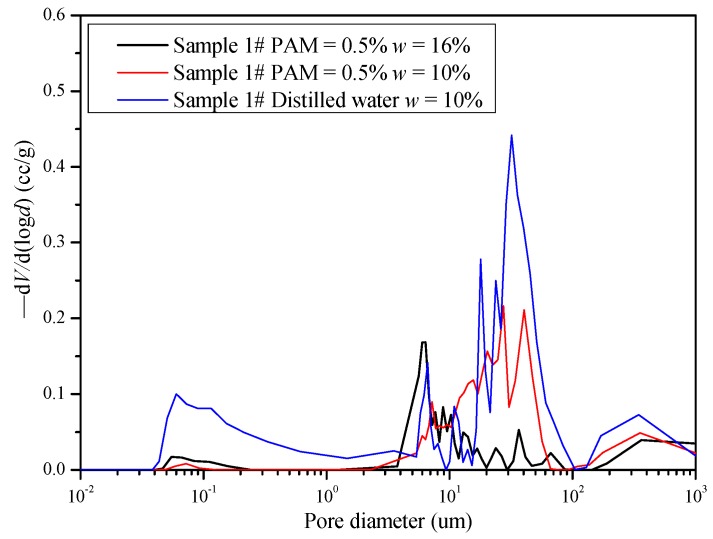
Pore size distribution as determined by mercury intrusion porosimetry (MIP) tests.

The PAM solution’s effect on the microstructure of the saline soil samples was examined by MIP and SEM at a magnification of 3000×. As shown in Figure 6, the pore diameter distribution of the 1# samples (samples from the crust), which were compacted at different PAM concentrations and with different water contents, were represented as *d* versus -dV/d(log*d*) curves, where *d* is the pore diameter. Hereafter, 1#-PAM0.5-W16 denotes a saline soil sample from the surface that was treated with a 0.5% PAM solution and compacted at an initial water content of 16%. The controlling pore diameter of 1#-PAM0.5-W16, which is on the wet side of the optimum water content, was around 7 μm. The controlling pore diameter of 1#-PAM0.5-W10, which is on the dry side of the optimum water content, was around 12 μm. A comparison between samples 1#-PAM0.5-W16 and 1#-PAM0.5-W10 showed that they both had a monomodel distribution, and the controlling pore diameter decreased as the water content increased in the PAM-treated soil. The pore diameter distribution of 1#-PAM0-W10 showed a typical bimodel curve, and the two peak values were 0.1 μm and 12 μm. A comparison between samples 1#-PAM0.5-W10 and 1#-PAM0-W10 indicates that the small pores could be filled by PAM.

**Figure 7:**
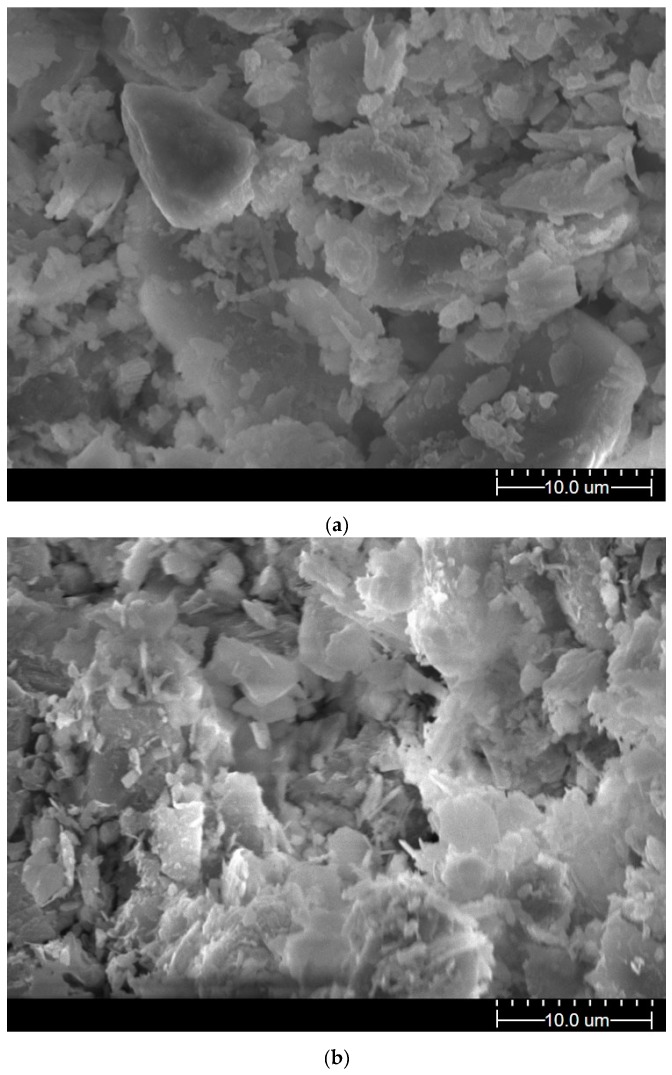
Scanning electron microscopy (SEM) images of soil samples (magnified 3000×): (**a**) 1#-PAM0-W10; (**b**) 1#-PAM0.5-W10.

The fabric elements of specimens 1#-PAM0-W10 and 1#-PAM0.5-W10 are shown in Figure 7. In Figure 7a, the particles in 1#-PAM0-W10 have a bulky pellet shape. In the compacted sample 1#-PAM0.5-W10, platelets that have a flaky and acicular shape were observed (Figure 7b). These results indicate that the micropores were filled by PAM agent during hydration. Thus, the macropores’ intensity decreased.

### 3.4. Cracking Behavior of the PAM-Treated Saline Soil Samples

**Figure 8:**
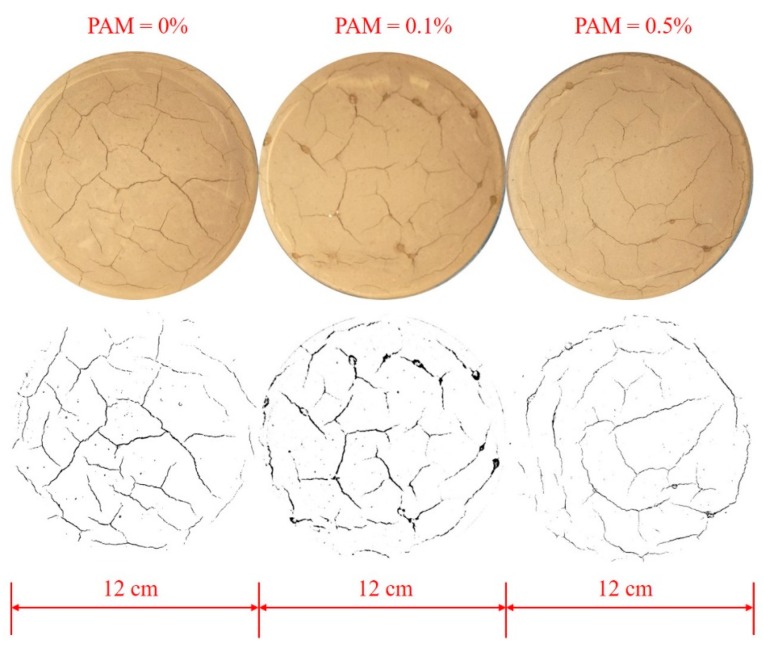
The surface crack images of saline soil samples from the surface.

**Figure 9:**
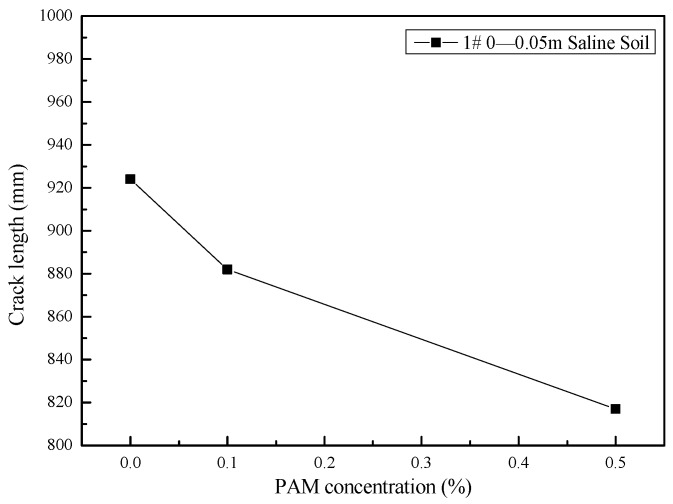
Crack length of saline soil samples from the surface.

Previous studies have found that a soil’s mass first decreases at a constant initial evaporation rate, then the mass loss subsequently slows during a falling evaporation rate stage, until the soil’s mass reaches a stable state [22,23]. In this study, the constant evaporation rate of both the untreated soil samples and the PAM-treated soil samples was 4.2 g per hour, and the soils cracked after six hours of evaporation. This indicated that influence of PAM concentration on the evaporation process for the surface saline soil samples was not significant. Figure 8 shows the typical crack pattern of the #1 samples after six hours of drying. The binary images show that the intensity of the network of cracks decreased as PAM concentration increased. According to the introduction of digital image processing techniques [24], the original photograph of the crack pattern was firstly changed to a grey level image. Secondly, due to the high contrast in grey level between cracks and aggregates, they were segmented into cracks and aggregates. This process was called binarization, resulting in binary black and white image. Thirdly, in order to determine the crack lengths, schematized structure of crack network was created by skeletonizing, which is defined as the middle line of crack segment. Finally, a further quantitative statistic of the length of skeleton was conducted. All these processes were operated automatically and conveniently in the software ‘Image J’ (National Institutes of Health, Research Triangle Region, ND, USA). The total crack length is illustrated in Figure 9. The results show that the total crack length in the surface saline soil sample decreased as PAM concentration increased (from 924 mm to 817 mm). Therefore, PAM’s stabilizing effect on saline soil under a wetting-drying cycle was confirmed.

## 4. Discussion

### 4.1. Compactibility and Physical Properties

A comparison between Figure 4 and Figure 5 shows that the maximum dry densities were lower for higher liquid limits, whereas the optimum moisture contents were close. A similar trend, based on Proctor compaction tests for 22 clayey soils (*w*_L_ = 20–70%), was reported by Blotz et al. (1998) [21].

It is widely accepted that the liquid limit is essentially a measure of the viscous resistance or shear strength of a soil as it approaches the liquid state [19]. Koumoto and Houlsby (2001) [25] analyzed the penetration mechanism of a fall cone into clay and suggested that the relationship between the water content *w* and the undrained shear strength *s*_u_ for a highly plastic remoulded clay is approximately linear on a double logarithmic scale over the entire water content range, from above the liquid limit to near the plastic limit. Consequently, the relationship between *w* and the cone penetration depth *h* is also linear on the double logarithmic plot. Sharma and Bora (2003) [26] experimentally verified that the undrained shear strength-water content relationship has been found to be log-linear for a wide range of water contents, starting from lower than the plastic limit to higher than the liquid limit.

Therefore, when the water content of two soils is close, the soil with the higher liquid limit will have a higher shear strength than the soil with the lower liquid limit. Figure 4 indicates that more water is needed for PAM-treated soil to disperse when it approaches a particular shearing resistance, and the undrained shear strength at a similar water content increases as the plastic limits and PAM concentration increase. From the Proctor compaction test results, the deformation is an instantaneous response to a reduction in weight, and the particles’ movement is mainly controlled by the soil’s undrained shear strength. During the compaction process, and under a certain compaction energy, a higher shearing resistance partially prevented the soil’s compression and dissipated some of the energy that makes the particles approach one another. As a result, the maximum dry densities of the #1 and #2 saline soil samples decreased as the PAM concentration increased to close to the optimum moisture content. This indicates that PAM-treated soil has potential as a lightweight filling material.

The SEM results show that the PAM agent dispersed the bulky pellets and flaky and acicular platelets formed the soil’s structure. The micropores were filled, and the shearing resistance between particles increased.

### 4.2. Cracking Behavior and Physical Properties

Many factors, such as a boundary constraint, flaws in the soil, the drying rate, shrinkage strain during drying, temperature, the soil layer’s thickness, and wetting and drying cycles, influence a soil surface’s cracking pattern. Generally, when the increase in tensile stress caused by desiccation exceeds the tensile strength within the upper layer of the soil, desiccation cracks occur on the surface [18,23,24,27,28,29,30,31,32,33,34,35].

According to previous studies, the drying process can result in the development of suction in the upper layer due to meniscus surface tension effects [35]. This increase in suction leads to volumetric shrinkage and a new arrangement of soil particles [36,37]. Thus, particles approach each other under volumetric shrinkage. Under a boundary constraint condition, tensile stress develops within the soil when it is restrained against shrinkage. If the initial water content, temperature, and boundary constraint are unified, soils with higher plasticity generally have a higher volume of water and are thus more prone to large volumetric shrinkage strains during drying [30,38,39]. Tang et al. (2008) [24] proposed that the crack intensity factor (CIF), which is the ratio of the crack area to the total surface area of a drying soil mass, is related to the soil fineness fraction, i.e., the more fine the content, the higher the obtained CIF.

As shown in Figure 7, a lower crack length was found for PAM-treated saline soil. This indicates that the PAM solution can aggregate fine particles. As indicated in Figure 4, an increase in the tensile strength between particles in PAM-treated soil reduced the shrinkage strain and the total crack length.

Macrocracks normally develop with the growth of microcracks, which is related to the inter-granular voids [40]. Costa et al. (2013) [41] identified that the flaws or pores within the material control crack initiation. As shown in Figure 6, a lower number of cross-points in the network of cracks in the PAM-treated saline soil were observed. This suggests that the number of flaws or pores within the material may decrease as PAM concentration increases. Thus, there are fewer initial microcracks and the crack tips develop a lower total crack length.

## 5. Conclusions

The consistency limits, compactability, and cracking behavior of PAM-treated saline soil from Gansu Province, China were investigated in this paper. The main conclusions are as follows:

(1) The liquid limits (LLs) and plastic limits (PLs) of saline soil from the surface (0–0.05 m) and at a depth of 2.0–3.0 m increased as PAM concentration increased (0.1–0.5% in mass ratio). Previous studies have verified that the LLs and PLs determined by cone penetration tests can be redefined in terms of their undrained shear strength. So, these results indicate that the undrained shear strength at a similar water content may be increased by increasing the PAM concentration.

(2) The maximum dry densities decreased as the PAM concentration and plasticity increased, and the optimum water contents of the two saline soil sample types did not significantly change. A comparison between liquid limits and compaction curves suggested that a higher shearing resistance partially prevented the soil’s compression and dissipated some of energy that causes the particles approach one another. Then, the maximum dry densities of the two saline soil sample types decreased as PAM concentration increased for a close to optimum moisture content. This indicates that PAM-treated soil has potential as a lightweight filling material. The SEM micrographs show that the PAM agent dispersed the bulky pellets, and flaky and acicular platelets formed the soil’s structure. The micropores were then filled.

(3) The quantitative analysis of crack patterns showed that the number of cross-points in the network of cracks and the crack length decreased as the PAM concentration increased. These results indicate that an increase in the tensile strength between particles reduces the shrinkage strain, and the number of flaws or pores within the material may decrease as the PAM concentration increases. Therefore, PAM’s stabilizing effect on saline soil under a wetting-drying cycle has been proven. Then, a potential use of polyacrylamide treated saline soil in embankment filling could be applied in practice. Besides, to clarify the economics of PAM usages, more tests should be performed by varying the PAM concentrations more finely in the future.

## Figures and Tables

**Table 1:** Physical properties of selected saline soil samples.

Soil Depth	Natural Water Content *w*_0_ (%)	Wet Density (Mg/m^3^)	Dry Density (Mg/m^3^)	Saturation Degree *Sr* (%)	Void Ratio *e*	Compression Coefficient *a*
0–0.05 m	12.2	1.86	1.66	60.4	0.5	0.1
2.0–3.0 m	27.1	2.05	1.61	100.0	0.8	0.4

**Table 2:** Chemical composition of two selected soil samples.

Soil Depth	PH	CO_3_^2−^ (mg/kg)	HCO_3_^−^ (mg/kg)	Cl^−^ (mg/kg)	Ca^2+^ (mg/kg)	Mg^2+^ (mg/kg)	Na^+^ + K^+^ (mg/kg)	Total Soluble Salt (mg/kg)	Salinity (%)
0–0.05 m	7.84	0	404.93	7407.77	4987.56	148.84	75.02	15,326.68	1.53
2.0–3.0 m	8.22	0	438.67	3037.36	337.27	55.81	3716.87	9913.03	0.99
